# Supplementary material for: Visual perturbation training to reduce visual dependency in Parkinson’s disease: A randomized controlled trial
Source: PLoS One. 2026 Mar 2;21(3):e0343223. doi: 10.1371/journal.pone.0343223 (PMC12952592; doi:10.1371/journal.pone.0343223)
Supplement: S1 Table — (DOCX) [file pone.0343223.s001.docx]

**Appendix 1: Self-reported (near)falls**

| Baseline falls | Month 1 | Month 2 | Month 3 | Month 4 |
| --- | --- | --- | --- | --- |
| **Control** | | | | |
| 3 | 0 | 0 | 0 | 0 |
| 0 | 0 | 0 | 0 | 0 |
| 5 | 1 | 1 | 1 | 0 |
| 1 | 0 | 1 | 1 | 2 |
| 1 | 0 | 0 | 0 | 0 |
| 90 | 14 | 5 | 4 | 1 |
| 0 | 0 | 1 | 0 | 0 |
| 0 | 1 | 0 | 0 | 0 |
| 0 | 0 | 0 | 0 | 0 |
| 0 | 0 | 0 | 0 | 0 |
| Average | | | | |
| 10 | 2 | 1 | 1 | 0 |
| **VPT** | | | | |
| 0 | 0 | 0 | 0 | 0 |
| 2 | 3 | 2 | 1 | 0 |
| 0 | 0 | 0 | 0 | 0 |
| 12 | 42 | 19 | 23 | 20 |
| 1 | 0 | 0 | 0 | 0 |
| 5 | 0 | 0 | 0 | 0 |
| 1 | 0 | 0 | 0 | 0 |
| 0 | 0 | 0 | 0 | 0 |
| 45 | 4 | 1 | 0 | 0 |
| 0 | 2 | 0 | 0 | 0 |
| 0 | 0 | 0 | 0 | 0 |
| 1 | 0 | 0 | 0 | 0 |
| 0 | 0 | 0 | 0 | 0 |
| 0 | 0 | 0 | 0 | 0 |
| Average | | | | |
| 5 | 4 | 2 | 2 | 1 |
