# Supplementary material for: Visual perturbation training to reduce visual dependency in Parkinson’s disease: A randomized controlled trial
Source: PLoS One. 2026 Mar 2;21(3):e0343223. doi: 10.1371/journal.pone.0343223 (PMC12952592; doi:10.1371/journal.pone.0343223)
Supplement: S3 Protocol — (DOCX) [file pone.0343223.s003.docx]

**Journal Requirements:**

When submitting your revision, we need you to address these additional requirements.

1**. Please ensure that your manuscript meets PLOS ONE's style requirements, including those for file naming. The PLOS ONE style templates can be found at**

[**https://journals.plos.org/plosone/s/file?id=wjVg/PLOSOne_formatting_sample_main_body.pdf**](https://journals.plos.org/plosone/s/file?id=wjVg/PLOSOne_formatting_sample_main_body.pdf) **and**

[**https://journals.plos.org/plosone/s/file?id=ba62/PLOSOne_formatting_sample_title_authors_affiliations.pdf**](https://track.editorialmanager.com/CL0/https:%2F%2Fjournals.plos.org%2Fplosone%2Fs%2Ffile%3Fid=ba62%2FPLOSOne_formatting_sample_title_authors_affiliations.pdf/1/010f019a9a5c5847-97e4193f-a02e-438c-a948-328dfb00b8c3-000000/BCQlow27Ky5uvOblVZnJCbUF6AuCNtSubRzWPYFEIvE=238)

The manuscript has now been reformatted to fit PLOS ONE’s style requirements.

**2. Thank you for submitting your clinical trial to PLOS ONE and for providing the name of the registry and the registration number. The information in the registry entry suggests that your trial was registered after patient recruitment began. PLOS ONE strongly encourages authors to register all trials before recruiting the first participant in a study.**

**As per the journal’s editorial policy, please include in the Methods section of your paper:**

**1) your reasons for your delay in registering this study (after enrolment of participants started);**

**2) confirmation that all related trials are registered by stating: “The authors confirm that all ongoing and related trials for this drug/intervention are registered”.**

This trial was registered before recruitment started. However, there was a typo in the first version of the submitted manuscript that may have caused some confusion (see previous mail from 13/08/2025). In the original manuscript, the start date was indicated to be January 9th 2022. However, this should have been January 9th 2023. The ethics approval was obtained in December 2022 and there was no amendment submitted to extend the originally planned recruitment period. The recruitment dates have been corrected in the manuscript.

**3. Thank you for stating the following financial disclosure:**

**“This study was funded by a grant from the Flemish Parkinson League (Vlaamse Parkinson Liga) and the King Baudouin Foundation (Koning Boudewijn Stichting), grant number 2022-J1811020-226020.”**

**Please state what role the funders took in the study.  If the funders had no role, please state: "The funders had no role in study design, data collection and analysis, decision to publish, or preparation of the manuscript."**

**If this statement is not correct you must amend it as needed.**

**Please include this amended Role of Funder statement in your cover letter; we will change the online submission form on your behalf.**

We have added the statement "The funders had no role in study design, data collection and analysis, decision to publish, or preparation of the manuscript." To the financial disclosure and removed all funding-related text from the manuscript (see comment 4).

**4. Please note that funding information should not appear in any section or other areas of your manuscript. We will only publish funding information present in the Funding Statement section of the online submission form. Please remove any funding-related text from the manuscript.**

See response to comment 3.

**5. In the online submission form you indicate that your data is not available for proprietary reasons and have provided a contact point for accessing this data. Please note that your current contact point is a co-author on this manuscript. According to our Data Policy, the contact point must not be an author on the manuscript and must be an institutional contact, ideally not an individual. Please revise your data statement to a non-author institutional point of contact, such as a data access or ethics committee, and send this to us via return email. Please also include contact information for the third party organization, and please include the full citation of where the data can be found.**

The anonymized VPT data has now been made available for publication with the manuscript and a new version of the availability statement has been uploaded.

**6. We note that Figure 1 includes an image of a participant in the study.**

**As per the PLOS ONE policy (**[**http://journals.plos.org/plosone/s/submission-guidelines#loc-human-subjects-research**](http://journals.plos.org/plosone/s/submission-guidelines#loc-human-subjects-research)**) on papers that include identifying, or potentially identifying, information, the individual(s) or parent(s)/guardian(s) must be informed of the terms of the PLOS open-access (CC-BY) license and provide specific permission for publication of these details under the terms of this license. Please download the Consent Form for Publication in a PLOS Journal (**[**http://journals.plos.org/plosone/s/file?id=8ce6/plos-consent-form-english.pdf**](http://journals.plos.org/plosone/s/file?id=8ce6/plos-consent-form-english.pdf)**). The signed consent form should not be submitted with the manuscript, but should be securely filed in the individual's case notes. Please amend the methods section and ethics statement of the manuscript to explicitly state that the patient/participant has provided consent for publication: “The individual in this manuscript has given written informed consent (as outlined in PLOS consent form) to publish these case details”.**

**If you are unable to obtain consent from the subject of the photograph, you will need to remove the figure and any other textual identifying information or case descriptions for this individual.**

The participant in Figure 2 did provide written consent for their image to be used for publication. However, as we were not able to get specific written consent for their picture to be published under the CC BY 4.0 license, the original has now been replaced by an avatar.

**7. If the reviewer comments include a recommendation to cite specific previously published works, please review and evaluate these publications to determine whether they are relevant and should be cited. There is no requirement to cite these works unless the editor has indicated otherwise.** 

**Additional Editor Comments**:

**I strongly reccomend:**

**improve the rationale of study showing that Individuals with visual impairment have often been observed to walk slower than individuals with unimpaired vision. This statement can be misplaced by typical low levels of physical activity and greater sedentary behavior in individuals with VI than the control population (e.g., PMID: 29614469).**

It is unclear why the effects of visual impairment would be relevant for this study, as it was not a part of the research question. Although all sensory systems can be affected by PD, the input from the vestibular and proprioceptive systems is generally affected to a much greater extent (As alluded to in reference 10). None of our participants reported suffering from visual impairments and, considering the size of the projection screen and nature of the perturbation, mild impairments would not have had any significant effect on the visual dependency scores. In fact, based on our data, their baseline gait speed was higher than their healthy peers. The authors feel that inclusion of this element may confuse readers with respect to the characteristics of the cohort tested in this study. However, the exclusion criteria may not have been clear enough on this point. They have now been amended to clarify visual impairments as a factor:

*“Due to the visual nature of the perturbations, candidates with severe visual impairments were also excluded. However, considering the size of the projection screen, light (PD-related) visual impairments were not deemed to hinder participation or affect outcomes. Participants were allowed to wear their daily visual aids at all times.”*

**Small sample size (reviewer 1),**

The issue with multiple comparisons in combination with the sample size has now been addressed. See reply to reviewer #1.

**Steps taken to mitigate this risk potential for bias during data collection or processing (reviewer 2)**

The clarification on procedures to limit risk of bias in data collection and processing have been addressed in our reply to reviewer #2.

**Consider including primary references where the gait abnormalities were observed in PD for example, (PMID: 33436993)**

- **Consider including the phatophysiological mechanism of these restrictions in PD.**
- **Particularly, I suggest trying to use one parameters very useful to analyze the functional mobility. Consider applying the rehabilitation locomotor index. To do this, you need just the walking speed and the lower limb length (great trochanter to the ground) or 0.54 of height (**[**https://www.ncbi.nlm.nih.gov/pmc/articles/PMC2872302/**](https://www.ncbi.nlm.nih.gov/pmc/articles/PMC2872302/)**)**
- **After, you need to apply these two simple equations: OWS (optimal walking speed, in m/s) = sqrt ( 0.25 x 9.81 x lower limb length (or 0.54 of height)) LRI (locomotor rehabilitation index, in %) = 100 x walking speed / OWS. The message is obtain the walking speed normalized based on theory of dynamic similarities and given an index that represents how is the person is close to your more economical metabolically to his/her optimal walking speed and where the pendular mechanism is more optimized. To understand in depth, please read:**[**http://www.clinicaltdd.com/text.asp?2016/1/2/86/184750**](http://track.editorialmanager.com/CL0/http:%2F%2Fwww.clinicaltdd.com%2Ftext.asp%3F2016%2F1%2F2%2F86%2F184750/1/010f019a9a5c5847-97e4193f-a02e-438c-a948-328dfb00b8c3-000000/C2_ZfGs8DGVG9fF8_1zX74S5hxvShv4arf1-P_ouQ9k=238)
- **This parameter was already used in normal (**[**https://doi.org/10.1016/j.gaitpost.2021.09.191**](https://track.editorialmanager.com/CL0/https:%2F%2Fdoi.org%2F10.1016%2Fj.gaitpost.2021.09.191/1/010f019a9a5c5847-97e4193f-a02e-438c-a948-328dfb00b8c3-000000/2_hNX9CXTLjFtKe08Jg6QgHqLMLpMA-yL34BwrzqyTA=238)**) and CHF (**[**https://pubmed.ncbi.nlm.nih.gov/23059867/**](https://track.editorialmanager.com/CL0/https:%2F%2Fpubmed.ncbi.nlm.nih.gov%2F23059867%2F/1/010f019a9a5c5847-97e4193f-a02e-438c-a948-328dfb00b8c3-000000/CaU-QEbOPED1M-La6gQkxy7fRepymT_KwoJ7Do5kdbg=238)**), Parkinson (**[**https://pubmed.ncbi.nlm.nih.gov/26833853/**](https://pubmed.ncbi.nlm.nih.gov/26833853/)**) individuals.**

We thank the editor for their constructive feedback and suggestions about providing a pathological reference frame for our findings on gait speed. We acknowledge that the rehabilitation locomotor index parameter can be a useful tool to assess the impact of PD on gait performance and to track disease progression over time. However, as stated in the discussion, the average gait speed of the participants in this study was similar to that of a healthy age-matched peer group and above the expected healthy gait speed of 1.0-1.2 m/s for this age group. As such, assessments of change in gait parameters during steady state gait were considered secondary outcomes. Consequently, recalculating the walking speed into a rehabilitation locomotor index would be unlikely to yield crucial new insights into the effects of VPT on gait. However, we will take this into consideration for future analyses of samples with pathological gait impairments.

**Reviewers' comments:

Reviewer's Responses to Questions**

**Comments to the Author**
**Reviewer #1: This was a simple parallel design comparison consisting of a randomized controlled trial of early-to-mid-stage people with Parkinson’s disease randomly assigned to a visual perturbation group or treadmill training-only control group. The study was designed appropriately with sample size and power considerations. There was much information presented and the paper was well presented, statistically. There were some minor concerns as noted below.
Interaction effects were analysed using mixed ANOVAs. Tests for normality were performed appropriately as needed.. When significant interaction effects were found, additional within-group post-hoc tests were performed using either paired samples t-tests or Wilcoxon signed-rank tests. For visual dependency, additional analyses were performed using data from the VPT group in order to gain insights on factors that may identify responders and non-responders. The analyses were appropriately selected and executed for this investigation.
The total sample size required was about 20 individuals. The final sample was 25. The sample size was small and the number of endpoints, especially in the gait category, was large. Thus the issue of multiple comparisons should have entered as a possible consideration and should be included in the limitations remarks. They do note that post-hoc tests revealed significant decreases in main effects of step time, stride time, stance time and swing time and increased cadence ( all p<0.001) which, if adjusting for multiple testing, would be significant. VD regression and correlation which were primary were sufficiently discussed. Falls were summarized as best as possible. Also, the limitations were clearly presented.**

We thank the reviewer for their positive evaluation of the manuscript. As stated, the sample size was relatively small but justified. The reviewer makes a valid point with regards to the absence of a correction for multiple comparisons. We did apply a BH correction to the results of the repeated measures ANOVA, which lead to the loss of all significance in changes of the steady-state gait parameters. However, we did find remarkably high effect sizes for all outcomes that were significant before correction and these all reached minimal detectable change thresholds. This, combined with the fact that the gait-based parameters were a secondary outcome and that the nature of the study was explorative, it was decided to report the uncorrected results. This has now been clarified in the methodological considerations and limitations section:

*“Third, a Benjamini-Hochberg correction for multiple testing was initially applied to the outcomes of the repeated measures ANOVA. Although this did not impact visual dependency, significance of all gait adaptations during steady state walking rose above the critical level (p = 0.05). However, in light of the explorative nature and relatively small sample size of this study, and taking into consideration that the uncorrected gait parameters that showed significant improvement all exceeded the minimal detectable change threshold with relatively large effect sizes (η^2^ > 0.14), the uncorrected results were reported instead.”*

**Reviewer #2: This manuscript by Baggen et al. presents a well-designed and timely randomized controlled trial investigating the effects of Visual Perturbation Training (VPT) on visual dependency and gait in individuals with Parkinson's Disease (PwPD). The study addresses an important clinical problem, falls in PD, by targeting a potential underlying mechanism: increased visual dependency. The application of virtual reality for gait rehabilitation is a promising and innovative approach. The methodology is generally robust, with a clear protocol, appropriate outcome measures, and sound statistical analysis. The results are promising, showing a significant reduction in visual dependency and improvements in temporal gait parameters in the VPT group compared to treadmill training alone.**
**While the study is strong, there are several points that require clarification and discussion to strengthen the manuscript and its impact.**
**Major Comments**
**Sample Size and Generalizability: The study successfully recruited a sample size that met its a-priori power calculation. However, the final sample of 25 participants (14 VPT, 11 CONT) is relatively small, which limits the generalizability of the findings and the power of subgroup analyses (e.g., responder vs. non-responder). The authors correctly acknowledge this. Furthermore, the cohort was highly functional (high baseline gait speed, no regular freezers, relatively young), which may not be representative of the broader PD population at risk for falls. The discussion of a potential ceiling effect and recruitment bias is appropriate, but the authors should more explicitly state how these factors limit the generalizability of their conclusions.**

We thank the reviewer for their positive and constructive feedback. Additional clarification on limitations of generalizability towards efficacy and feasibility in more sedentary and impaired PwPD has now been added to this section:

*“...indicated a relatively high level of physical fitness in our cohort [39] which may not be representative of the entire PD population. As such, even though a scalable progression program was put in place a-priori, and even ‘non-responders’ completed the entire program, the generalizability of these results towards efficacy and feasibility of VPT in more sedentary and impaired PwPD remains limited.”*

**Definition and Measurement of Visual Dependency: The concept of "visual dependency" is central to the study, but its operationalization could be explained more clearly for a broader audience.**

Additional explanation on quantification of visual dependency was added to the introduction:

*“Visual dependency is defined as a “reduced ability to disregard visual cues in complex or conflicting visual environments” [8], and can be quantified by measuring changes in gait following visual cues or perturbations. Unreliable visual cues and perturbations can result in visual-vestibular mismatching of perceived and actual self-motion, subsequently inhibiting selection of appropriate motor strategies for balance control, and potentially leading to impaired balance control during gait and increased fall risk [8]. As such, quantification of visual dependency can be used to assess the level of visual control during posture and equilibrium, and help to identify PwPD at risk [15]. Subsequently, this information may be used to tailor rehabilitation programs in clinical practice.”*

The specific definition (quantification method) of visual dependency for this study was provided in the previous version of the manuscript in the last paragraph of section ‘visual dependency’ in the methods and in table 1:

*“Then, cross-correlations were calculated between screen and ML-CoM movements, to determine the lag of ML-CoM movements relative to the movements of the projected environment at which the correlation between the two signals was highest. The correlation and regression coefficient between these two signals (screen rotation and lag-corrected ML-CoM movement), were calculated as measures for visual dependency.”*

**The authors use the correlation and regression coefficient between screen rotation and ML-CoM movement. A high correlation indicates that the CoM movement closely follows the visual perturbation, which is interpreted as high visual dependency. However, it would be helpful to discuss what a "good" or "healthy" value for this metric is. Is the goal a correlation of zero?**

Unfortunately, due to the novelty of this application and concurrent method of quantification of visual dependency, no norm data for visual dependency in healthy participants is currently available. Because, even in healthy systems there will always be a weighted visual input, a correlation of zero is unlikely. Moreover, in this task it would suggest perfect anti-phase movement with the perturbation provided. We may speculate that a correlation of 0.5 is a good rehabilitation goal, but this cannot be confirmed with our current dataset and would require a study with much larger sample sizes. Therefore, we decided to omit this from the discussion. This has now been clarified in the methodological considerations and limitations section:

*“Due to the novelty of these methods for testing visual dependency, no reference data for good or healthy performance, or minimal clinically important differences in PwPD are currently available. In this study, visual dependency was quantified as the correlation between visual perturbation and ML-CoM signals. Consequently, a correlation of zero would imply perfect anti-phase movement to the perturbations and thus an inverse relationship. As such, it is reasonable to speculate that correlations around 0.5 should be considered the target for rehabilitation. However, even in healthy systems, there will always be a weighted effect of visual input on ML-CoM movement. Therefore, reference data from larger healthy cohorts is needed to assess the efficacy and minimally clinically important effect of VPT in PwPD.”*

**The authors mention that the reduction in visual dependency post-VPT is not merely a phase shift, as the time-series shapes become more dissimilar (Fig 3). This is a crucial point supporting the sensory reweighting hypothesis. This interpretation should be emphasized and discussed in more depth in the results and discussion sections.**

We agree that this point would benefit from additional emphasis. We have addressed this by a more detailed assessment of Figure 3 in the Results section:

*“This figure illustrates that, although there is clearly still an effect of the visual perturbation on general ML-CoM movement, decoupling is visible as the pattern and regularity of the ML-CoM become more dissimilar from pre- to post-intervention.”*

...and by describing the implications of changes in visual dependency (as defined in this study) for assessing visual downweighting following findings from Feller et al. 2019 in the Discussion:

*“As illustrated in Fig 3, following 11 bouts of VPT, the reduction in visual dependency appears to be not merely attributable to phase shifts in ML-CoM displacement since the shape of the time-series also shows a more irregular frequency and reduced coherence with the visual perturbation signal, especially during late adaptation. This change in ML-CoM coherence while maintaining dynamic balance in a visually challenging environment likely indicates down-weighting of unreliable visual cues following VPT, which is in line with findings from a previous study using a frequency response function and coherence obtained from a visual perturbation paradigm during stance in PwPD [44].*

**Clinical Significance and Falls: The primary outcome (visual dependency) is a laboratory-based measure. While it is a mechanistically interesting target, its direct clinical relevance needs further justification.**

We have now emphasized the clinical relevance of measuring visual dependency in the concurrent paragraph in the discussion:

*“The ability to quantify visual dependency and elicit sensory reweighting using visual perturbations during gait may provide a valuable tool in clinical practice to determine the level of visual control exerted during posture and equilibrium, and help to reduce fall risk in PwPD.”*

**The study found no significant effect on (near) falls. The authors provide plausible explanations (self-report inaccuracies, pooling of falls/near-falls, small sample). However, the conclusion that VPT improves "fundamental sensory and motor mechanisms associated with fall risk" would be stronger if a correlation (even if not significant in this small sample) between the reduction in visual dependency and the reduction in falls was tested and reported. This would help bridge the gap between the mechanistic outcome and the clinical endpoint.**

We appreciate the request to improve the link between the mechanistic assessments and clinical implications. However, we have to emphasize that this was not the primary goal for this study (and the design was likely not suitable to draw such conclusions). As such, (near-)falls were very much a preliminary outcome. The data were reported for the sake of transparency, but we feel that drawing conclusions without significant results or sufficient effect sizes would be misleading to the reader.

**Blinding and Potential Bias: The manuscript states that participants were blinded, but assessors were not. While the outcomes are objective and automated, the potential for bias during data collection or processing cannot be entirely ruled out. The authors should briefly discuss the steps taken to mitigate this risk (e.g., standardized scripts, automated data processing pipelines) to reassure readers.**

Clarification on standardization of both measurement scripts and automation of data processing has now been added to the relevant paragraph of the limitations section:

*“...as well as the data processing pipelines were identical for all participants...”*

***Minor Comments

Abstract: The abstract is clear but could briefly mention the specific patient population's characteristics (e.g., "early-to-mid-stage PwPD without regular freezing of gait") to provide better context.***

This has now been added to the abstract. **Methods: Please clarify the duration of the "four-week baseline period." It is mentioned that this was a period of "usual care" before the 6-week training started. Was any data collected at the end of this baseline period to confirm stability, or was the pre-test done immediately before training initiation?**

The authors acknowledge that it may not have been clear that this only served to check baseline fluctuations in visual dependency (as this was a novel outcome). We have now specified that this was the only outcome tested after the 4-week baseline period:

*“This baseline period allowed to control for non-training related fluctuations in visual dependency only.”*

**Results: The criteria for defining "responders" (≥10% reduction) is described as "arbitrary." While pragmatic, could this be justified by reference to a Minimal Clinically Important Difference (MCID) or similar concept, even if from a different field? Alternatively, a median split or regression-based approach could be considered, though the small sample size makes this challenging.**

As with the omission of a ‘target’ for rehabilitation, the authors chose to emphasize that this threshold was arbitrary due to the lack of reference data (even from different fields or pathologies), related to the novelty of this method for defining visual dependency. The authors feel that this may be misleading for clinicians. This has now been clarified in the Methodological considerations and limitations section:

*“Due to the novelty of these methods for testing visual dependency, no reference data for good or healthy performance or minimal clinically important differences in PwPD are currently available…reference data from larger healthy cohorts is needed to assess the efficacy and minimally clinically important effect of VPT in PwPD.”*

**Discussion: The discussion is thorough. The speculation on why spatial parameters did not change (e.g., unaffected motor strategies) is interesting. It would be valuable to also briefly hypothesize why VPT would be expected to change spatial parameters, to give the reader a fuller picture of the theoretical framework.**

Calabro et al. (ref 33) specifically attributed the changes in step length and width to more rapid swing phases as a result of more stable stance phases. This has now been added to the discussion:

*“…attributed to more rapid swing phases as a result of more stable stance phases [18, 32, 33].”*

**Data Availability Statement: The current statement, "available from the corresponding author upon reasonable request," does not fully comply with PLOS ONE's data policy, which requires unconditional public availability. The authors should deposit their data in a public repository (e.g., Figshare, Dryad) and provide the DOI/accession number, or provide a robust justification for any restrictions.**

We have now added the anonymised data in the supporting materials for this manuscript.

**In summary, this is a valuable and well-executed study that provides initial evidence for the efficacy of VPT in reducing visual dependency in PwPD. The manuscript is well-written, and the experimental design is sound. Addressing the points above, particularly regarding the clinical translation of the visual dependency metric and the generalizability of the findings, will significantly strengthen the manuscript and its contribution to the field.**

We thank the reviewers for their constructive feedback and hope the amendments made to the manuscript addressed your suggestions and concerns appropriately.
